# Intranodal palisaded myofibroblastoma (intranodal hemorrhagic spindle cell tumor with amianthoid fibers): a case report and literature review

**DOI:** 10.1186/1746-1596-5-12

**Published:** 2010-02-09

**Authors:** Nilüfer Onak Kandemir, Figen Barut, Turan Ekinci, Çetin Karagülle, Şükrü Oğuz Özdamar

**Affiliations:** 1 Department of Pathology, Faculty of Medicine, Zonguldak Karaelmas University, Zonguldak, Turkey; 2 Department of Pathology, Atatürk State Hospital, Zonguldak, Turkey

## Abstract

Intranodal palisaded myofibroblastoma (IPM) is a benign mesenchymal neoplasm originating from smooth muscle cells and myofibroblasts. It is characterized by spindle cells, amianthoid fibers, and by the proliferation of hemosiderin-containing histiocytes in the lymph node. A nodular lesion was excised from the inguinal region of an 80-year-old male patient. Macroscopic examination of a section of the lesion demonstrated a solid appearance with hemorrhagic areas. Microscopic examination revealed spindle cell proliferation, amianthoid fibers, hemosiderin pigment, and extravasated erythrocytes. Nuclei of the spindle cells displayed a palisaded appearance. Compressed lymphoid tissue was observed around the lesion. With Masson's trichrome, spindle cells stained as smooth muscle, whereas collagen staining was observed in homogeneous eosinophilic accumulations. Neoplastic cells were identified by the presence of vimentin and SMA. The Ki67 index was less than 1%. In light of these results, the case was diagnosed as "intranodal palisaded myofibroblastoma." IPM is an uncommon neoplasm originating from the stromal component of the lymph node. Although IPM is benign, it is frequently confused with metastatic lesions.

## Background

Intranodal palisaded myofibroblastoma (IPM), also known as intranodal hemorrhagic spindle-cell tumor with amianthoid fibers, is a benign mesenchymal neoplasm originating from differentiated smooth muscle cells and myofibroblasts. It is characterized by the proliferation of hemosiderin-laden histiocytes, spindle cells, and amianthoid fibers in the lymph node [1-3]. Genetic evidence associated with viral agents, and excessive expression of cyclin D1, suggest that viral oncogenesis may be involved in the etiology of these lesions [4-6]. To date, approximately 50 cases have been reported in the literature. Although its behavior is benign, IPM is frequently confused with metastatic lesions, making correct identification important. In differential diagnosis, Kaposi sarcoma (KS) and schwannoma, as well as benign and malignant spindle-cell neoplasms, should be considered [1-3]. In the present study, a case of IPM identified in an 80-year-old male patient is discussed, with regard to the pathogenesis of the tumor as well as to its histological and immunohistochemical characteristics.

## Case presentation

### Clinical characteristics

The physical examination of an 80-year-old male patient, who presented with a 1-year history of a growing mass in the left inguinal region, revealed a painless nodular lesion with an elastic consistency. The lesion was clinically evaluated as lymphadenopathy and excised. The patient was discharged without complications and has been free of disease for 1 year.

### Macroscopic and microscopic findings

Grossly, the round lesion, 1.5 × 1 × 1 cm in size, had a tan, solid cut surface, with patchy red-brown areas. The lymph node was fixed in 10% formaIin and embedded in paraffin. Five-micrometer sections were stained with hematoxyIin-eosin (H&E) and Masson's trichrome (MT) stains. Immunohistochemically, the sections were stained with antibody against vimentin, muscle-specific actin (SMA), desmin, S-100, CD-117, Factor XIIIa, CD34, CD31, CD68, HMB45, keratin, epithelial membrane antigen (EMA), Ki-67, cyclin D1, and human herpesvirus 8 (HHV-8) using the avidin-biotin-peroxidase complex (ABC) technique.

**Figure 1:**
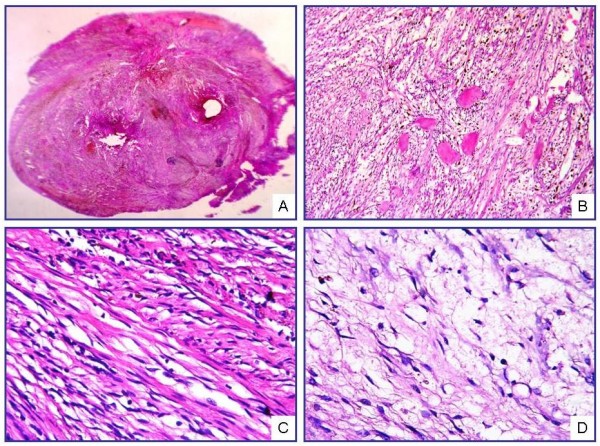
**Microscopic view of IPM under low magnification (A)**. Cellular area formed by spindle cells and amianthoid fibers (B, C). Myxoid changes and hypocellular areas with marked edema (D) (H&E; A, ×40; B, ×200; C, D, ×400).

**Figure 2:**
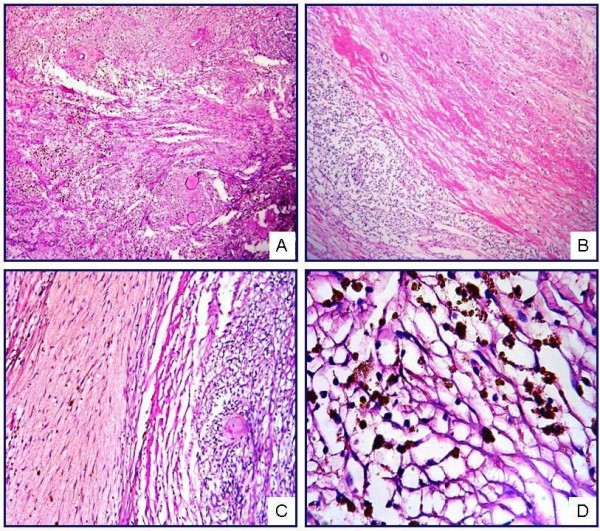
**IPM showing short fascicles of spindle cells and amianthoid fibers (A)**. The lesion contains compressed lymphoid tissue (B), intraparenchymal hemorrhage (C), and hemosiderin pigments, both free and phagocytosed by histiocytes (D) (H&E; A, ×50; B, ×50; C, ×100; D, ×400).

Microscopic examination showed spindle-cell proliferation alongside homogeneous eosinophilic accumulations, hemosiderin-laden macrophages, and extravasated erythrocytes. The nuclei of the spindle cells displayed a patchy pattern of palisading. Those cells were observed to have scant eosinophilic cytoplasm, elongated nuclei, and a coarse chromatin pattern. Among the spindle cells, collagen accumulations were recognized as "amianthoid fibers" exhibiting an irregular distribution and forming stellate structures in some areas (Fig. 1). The lesion demonstrated diffuse old and fresh hemorrhagic findings. No atypia, mitosis, or necrosis was identified in the cells forming the lesion, which was surrounded by compressed lymphoid tissue and a fibrous capsule (Fig. 2).

### Histochemical and immunohistochemical findings

**Figure 3:**
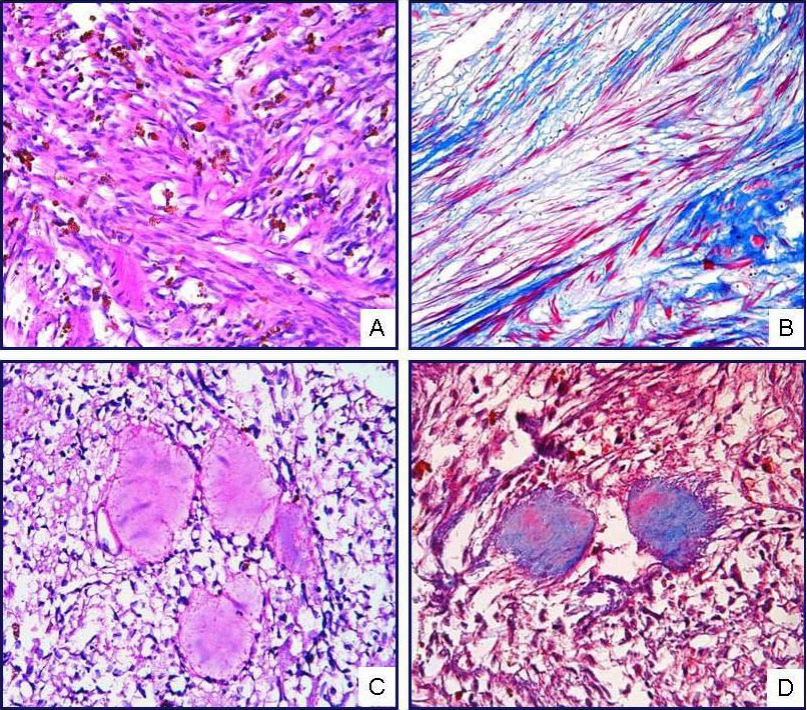
**(A) Hemosiderin pigment with irregular distribution is observed among the spindle cells forming interlacing bundles (H&E, ×400)**. (B) Spindle cells showed a staining with MT in favor of muscle (MT, ×400). (C) Acellular material accumulation with homogeneous eosinophilic appearance, called as "amianthoid fibers," is observed in the lesion (H&E, ×400). (D) Amianthoid fibers demonstrated staining typical of collagen (MT, ×400).

**Figure 4:**
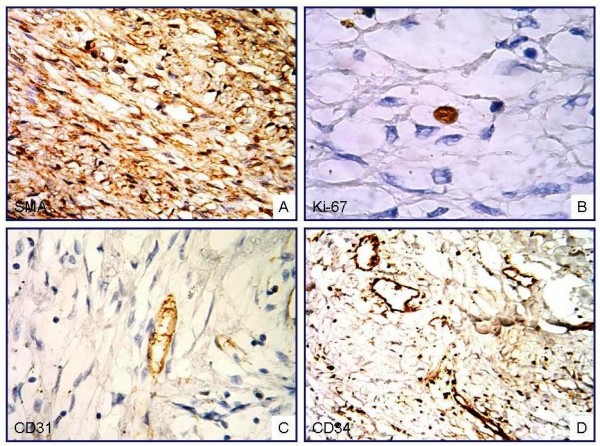
**Diffuse positive immunoreaction with antibodies against SMA (A) and focal positive Ki-67 immunoreaction (B) are observed in the spindle cells**. No immunoreaction was observed for CD31 (C) and CD34 (D) in neoplastic cells (ABC-DAB; A. ×200; B, C, ×400; D, ×200).

MT staining identified the spindle cells as smooth muscle, whereas the homogeneous eosinophilic accumulations were found to be positive for collagen (Fig. 3). Neoplastic cells displayed a positive reaction for vimentin and SMA, and a negative reaction for desmin, S-100, CD-117, Factor XIIIa, CD34, CD31, CD68, HMB45, EMA, and keratin. The Ki-67 proliferative index was below 1% (Fig. 4). Cyclin D1 and HHV-8 antibodies produced no immunoreaction. In light of these results, the case was diagnosed as "intranodal palisaded myofibroblastoma."

## Discussion

IPM is a rare neoplasm arising from the stromal component of the lymph node. Almost all cases develop in lymph nodes of the inguinal region. Although uncommon, it may also be seen in submandibular and mediastinal lymph nodes [7,8]. Initially, it was defined as a malignant neurilemmoma, first by Deligdish et al in 1968 [9], and later by Katz et al in 1974 [10]. In 1989, three groups of investigators described this tumor, giving it different names. Weis et al proposed the name "palisaded myofibroblastoma" [1], whereas Suster et al and Lee et al described it as "intranodal hemorrhagic spindle-cell tumor with amianthoid fibers" and "solitary spindle-cell tumor with myoid differentiation of the lymph node," respectively [2,3]. Currently, the term "intranodal palisaded myofibroblastoma" is preferred because it reflects the myofibroblastic origin of the tumor, as well as its histomorphological composition of prominent palisaded spindle-cells, and its localization to lymph nodes.

IPM produces a slow-growing, painless inguinal mass. Numerous cases are seen among men between 45 and 55 years of age. Grossly, the section of the lymph node has a solid appearance and displays irregular hemorrhagic areas. Histopathologically, it is characterized by various microscopic properties. In most cases, the tumor is observed with lymphoid tissue compressed to the periphery of the lesion. Spindle cells with nuclear palisading, intraparenchymal hemorrhage and erythrocyte extravasation, collagen deposits called amianthoid fibers, and intracellular or extracellular bodies reacting with smooth-muscle actin (SMA) and vimentin, are all seen in these lesions. Immunohistochemically, the spindle cells are positive for SMA and vimentin. No reaction is observed with many other antibodies. The tumor cells exhibit a low proliferative index [1-3].

Weis proposed that IPM originated from the walls of blood vessels in the lymph node [1]. The fact that inguinal lymph nodes have richer vascular components supports this claim. Moreover, immunohistochemical and electron microscopic findings have shown similarities between these lesions and the myofibroblasts and/or smooth muscle cells of blood vessels. Kleist et al demonstrated cyclin D1 expression in 50% of spindle cells in these lesions [4]. Genetic studies conducted with the same cases did not show any mutations in the CCND1 gene locus. These investigators suggested that cyclin D1 over-expression develops independently from any genetic changes and influences the spindle cells as a growth factor [4]. Genetic evidence associated with HHV-8 and Epstein - Barr virus (EBV) suggests that a viral mechanism of oncogenesis might be involved in the development of these lesions [5,6].

Typically, IPM is composed of spindle cells. Therefore, pathologic differential diagnosis should comprise the large diagnostic spectrum in which spindle cells are involved. Malignant spindle-cell tumors and non-IPM benign mesenchymal lesions of the lymph node must be taken into account. To diagnose as IPM, non-IPM tumors or pseudotumors must be excluded clinically, histomorphologically, and immunohistochemically. Neoplasms that have spindle-cell morphology can originate from vascular endothelium, fibroblasts, histiocytes, smooth muscle cells, myofibroblasts, dendritic reticulum cells, or interdigitating reticulum cells, all of which are stromal components of lymph nodes. These rarely seen lesions can imitate IPM histologically.

Schwannoma and KS are the most important tumors in the differential diagnosis. Moreover, hemangioendothelioma, dendritic reticulum cell tumor, and inflammatory myofibroblastic tumor (IMT) should also be considered in the differential diagnosis [1-3,11].

Schwannomas, extending through the peripheral nerve sheath, contain spindle cells with nuclear palisading and are often confused with IPM. Since it lacks peripheral nerve innervation, the lymph node is not expected to be a site of schwannoma development. Immunohistochemically, detection of S-100 immunoreaction contributes to the differential diagnosis of those two lesions.

Because IPM contains extravasated erythrocytes and hemosiderin pigments among the spindle cells, it may be mistaken for KS. In some IPM cases, detection of HHV-8 immunoreactivity reduces the utility of this marker in the differential diagnosis. However, histomorphologically, nuclear palisading and amianthoid fibers are not observed in KS. Moreover, the absence of immunoreaction with endothelial markers also supports an IPM diagnosis [12,13].

IMT is characterized by proliferation of spindle cells in the lymph node, a vascular structure laid down by compressed endothelium, and a dense inflammatory cell component. That the amianthoid fibers observed in IPM are not present is very helpful for differential diagnosis, and an inflammatory component rich in plasma cells is included in these lesions, which otherwise have similar immunohistochemical profiles to IPM [12].

IPMs are well differentiated tumors with low proliferative activity. They have been agreed to have a benign biological behavior. For this reason, distinguishing them from primary and metastatic malignant lesions of the lymph node is important. Malignant spindle-cell tumors can infiltrate lymph nodes by direct invasion or by metastasis. Primary and metastatic sarcomas of lymph node can be recognized by their marked pleomorphism and atypia, high mitotic activity, and by the expression of features characteristic of the cells of origin.

Spindle-cell melanoma is another malignant tumor that must be taken into account in differential diagnosis. Hemosiderin pigment, which is frequently seen in IPM, can be confused with melanin histologically. It contributes to the differential diagnosis that in melanomas, S-100 and HMB-45 immunoreactivity are seen in spindle cells. Unlike IPM, carcinomas express epithelial indicators (i.e., keratin, EMA) show marked atypia and pleomorphism and have high proliferative activity [11-14].

Since IPM forms a mass in the inguinal region, cytological samples can be obtained. Martinez et al described the cytological characteristics of these lesions as spindle cells with no nuclear atypia on smears with moderate cellularity, fibrillar background material, and hemosiderin granules and hemosiderin-laden histiocytes [15]. The biological behavior of these lesions is benign. In the literature, the local recurrence rate is about 6%, and no malignant transformation has been reported [1-3,12-15]. Regarding treatment, total removal of the lesion suffices.

Our case was a male patient at an advanced age, presenting with an IPM in the inguinal region, the typical location for that tumor. This painless and slow-growing lesion was evaluated to be clinically consistent with lymphadenopathy, and was excised with the prediagnosis of lymphoma. Microscopic diagnostic features of IPM, such as spindle cells with nuclear palisading, amianthoid fibers, hemosiderin pigment, and extravasated erythrocytes, were observed in this case. Residual lymphoid tissue was found to be compressed to the periphery of the lesion. Histochemically, amianthoid fibers showed staining for collagen, whereas the spindle cells stained as muscle. Immunohistochemically, spindle cells displayed a positive reaction for SMA. Our findings support the suggestion that these tumors arise from differentiated smooth muscle or myofibroblastic cells. In our case, no immunoreaction with cyclin D1 or HHV-8 was observed.

In conclusion, IPM, a rare tumor, should be considered in differential diagnosis because of its ability to mimic metastatic lesions in the lymph node. Future studies supported by molecular techniques and performed on large tumor series should further explain the pathogenesis of those lesions.

## Abbreviations

IPM: intranodal palisaded myofibroblastoma; KS: Kaposi sarcoma; H&E: hematoxyIin-eosin; IMT: inflammatory myofibroblastic tumor; SMA: muscIe specific actin; HHV-8: human herpesvirus 8; ABC: avidin-biotin peroxidase complex; EBV: Epstein-Barr virus; IHC: immunohistochemistry; MT: Masson Trichrome.

## Competing interests

The authors declare that they have no competing interests.

## Authors' contributions

NOK and FB performed microscopic evaluation, conducted the design of the study and drafted the manuscript. ÇK and ŞOÖ participated in histological evaluation and the design of the study. TE participated in immunohistochemical evaluation and helped to draft the manuscript. All authors read and approved the final manuscript.

## Consent

Written informed consent was obtained for publication of this case report and accompanying images. A copy of the written consent is available for review by the Editor-in-Chief of this journal.
